# Highly Complete Follow‐Up Data Are Necessary to Ascertain the Actual Complication Rate in Orchidopexy for Undescended Testes

**DOI:** 10.1111/iju.70168

**Published:** 2025-07-04

**Authors:** Mick Uijldert, Luitzen A. Groen, Keetje L. De Mooij, Michiel J. S. Oosterveld, Harrie P. Beerlage, Tom P. V. M. De Jong, Rogier P. J. Schroeder

**Affiliations:** ^1^ Department of Pediatric Urology Wilhelmina Children's Hospital, UMC Utrecht, Utrecht University Utrecht the Netherlands; ^2^ Department of Pediatrics Emma Children's Hospital, Amsterdam UMC, University of Amsterdam Amsterdam the Netherlands; ^3^ Department of Pediatric Urology Emma Children's Hospital, Amsterdam UMC, University of Amsterdam Amsterdam the Netherlands

**Keywords:** complete follow‐up, complications, orchidopexy, surgery outcomes, testis

## Abstract

**Objective:**

This study aimed to assess orchidopexy outcomes in a tertiary pediatric urology center.

**Methods:**

A retrospective review of orchidopexies at our institution initially showed a 20% follow‐up loss. To ensure more reliable outcomes, patients and parents were actively contacted and encouraged to attend follow‐up visits 6 months post‐surgery, reducing follow‐up loss and enhancing the accuracy of our data.

**Results:**

Two hundred eleven patients with 254 testicular units were included, with only five patients lost to follow‐up due to relocation, yielding a follow‐up rate of 98%. The median follow‐up time was 7 months. Of 206 inguinal orchidopexies, three were atrophied, and three had an inguinal position. Among 25 patients who underwent a (staged) laparoscopic procedure for abdominal testis, four were found to be atrophied. Of the 18 redo orchidopexies, one testis atrophied. One patient suffered a severe complication, Fournier's gangrene, due to streptococcus infection with uneventful healing after multiple surgeries.

**Conclusions:**

In a tertiary setting with a high follow‐up percentage and orchidopexy performed or supervised by a fellowship‐trained pediatric urologist, the success rate of standard orchidopexy was 97%, for redo orchidopexy 94%, and for laparoscopic (staged) procedures 80%. The reported outcomes can be used in more accurate patient/parental counseling in daily clinical practice and guidelines.

## 1 Introduction

Non‐scrotal testis (NST) is the most common congenital abnormality among boys, with a prevalence of 1%–8% among newborns [1, 2]. The prevalence is even higher among boys born prematurely or small for gestational age. Many NSTs descend after birth, but spontaneous descent is unlikely to occur after 6 months [3, 4, 5]. At the age of 1 year, 1% of boys still has non‐scrotal testes [6], yielding an incidence of NST of 20.000 per year in the European Union. If treatment is not performed timely, boys with NST have an increased risk of subfertility [7, 8] and neoplasia [8, 9]. Therefore, surgical correction before the age of 18 months is the current standard of care [10, 11, 12, 13].

Previous studies reporting results after orchidopexy have been limited by levels of loss to follow‐up ranging between 15% and 22% [4, 14, 15]. Such a large portion of patients might introduce selection bias. In a prior evaluation of boys operated for NST in our center, we observed a similar rate of loss to follow‐up. Henceforth, a concerted effort was made to achieve near‐complete levels of follow‐up of patients (aim: follow‐up rate of > 95%).

According to literature, the overall success rate of all orchidopexies (palpable and non‐palpable testes) is at least 88%, and 92% for inguinal orchidopexies (palpable testes) [8, 16]. Unsuccessful surgery may result in an incorrect position of the testis or testicular atrophy. We postulated that the surgeon's level of training might affect the outcome of orchidopexies.

For these reasons, we retrospectively reviewed all patients operated consecutively for NST at our institution over a period of 2 years. Goal of our study was to assess short‐term complication rates and postoperative outcomes of orchidopexies in a patient cohort with very low percentage of loss to follow‐up.

## 2 Methods

Data on all patients aged < 18 years who underwent orchidopexy at the pediatric urology department at Wilhelmina's Children Hospital UMC Utrecht, a tertiary care center, between January 2015 and December 2016 were analyzed retrospectively. Children with pre‐existent testicular atrophy and/or syndromic disorders were excluded. The latter to create a group as homogeneous as possible with less chance of intrinsic testicular tissue dysfunction.

The results of three surgical procedures for non‐scrotal testes were evaluated: firstly, inguinal orchidopexy for inguinal localized testes; secondly, laparoscopic (staged) Fowler‐Stephens procedures for high abdominal testes; and thirdly, redo cases. The indication for redo cases was a non‐scrotal position after surgery; in most cases, referred from a general hospital. All surgeries were performed or supervised by 2‐year fellowship‐trained pediatric urologists (FEAPU). Key points of the surgical techniques used are summarized in Table 1. The standardized surgical steps were combined with careful tissue handling.

**TABLE 1:** Key points of the surgical procedure.

Inguinal orchidopexy					
Step 1: Access: Incision at the level of the external inguinal ring. The right spot can be palpated through the skin by palpating the tubercle of the pubic bone.	Step 2: Identification of testis. The inguinal canal is preferably left untouched and should only be opened in case of a high position. Gubernaculum is divided. Gubernaculum can be used to grasp and mobilize the testicle without grasping it directly. Attachments are freed and the testicle is externalized.	Step 3: Opening of the external spermatic fascia on two Halsted arterial clamps or small mosquitoes. Opening of the internal spermatic fascia on two Halsted arterial clamps or small mosquito forceps. Remove any barriers to testicular descent such as internal spermatic fascia and, if present, an open processus vaginalis or inguinal hernia. Vessels and ductus can now be mobilized easily.	Step 4: A high scrotal incision is made. Dartos pouch is created bluntly with scissors. Testis is pulled through to the scrotal incision.	Step 5: Pull‐trough opening is narrowed by gently placing two approximation sutures cranially to the spermatic cord.	Step 6: Testis is placed in the Dartos pouch. Testis is left uncovered, no longer surrounded by tunica vaginalis, and will adhere to surrounding tissues.

Laparoscopic orchidopexy, 1st stage/single‐stage			
Step 1: Laparoscopic: open introduction of trocar according to Hasson at the umbilicus. Working ports as lateral as possible on the left and right iliac fossa. Placing of trocars too medially negatively impacts the working angle.	Step 2: If the testis can be brought towards the contralateral inguinal ring, a one‐stage procedure can be performed without ligation of the vessels. Continue with step 4, inguinal orchidopexy	Step 3: If the testis cannot be brought towards the contralateral inguinal ring, a two‐stage procedure must be performed with ligation of the vessels.	Step 4: 1st stage: spermatic vessels are ligated with two non‐absorbable sutures 3/0. Vessels are divided between the two sutures. A safe distance to the ductus is maintained and the ductus is not touched by any instrument. Continue to 2nd stage.

Laparoscopic orchidopexy, 2nd stage			
Step 1: Re‐laparoscopy is performed after > 6 months. The distal suture is grasped. A peritoneal flap is made around the testicle and around the ductus with a safe distance of 1–2 cm. The ductus is not touched directly with any instrument.	Step 2: The testis can be gently grasped. Grasping of the epididymis is avoided.	Step 3: A high scrotal incision is made. Firstly, a dartos pouch is made. Secondly, a mosquito is introduced into the abdominal cavity through the scrotal incision. The testis is preferably externalized medially to the epigastric vessels.	Step 4: This can be realized by introducing the mosquito under laparoscopic guidance. Testis can be pulled into the scrotal incision. Continue with step 5 inguinal orchidopexy.

All patients were reviewed at the outpatient clinic 6 months after surgery. In case of a no‐show, patients and parents were actively contacted to attend a rescheduled follow‐up visit. Success was defined as the scrotal position of the testis with no signs of testicular atrophy upon clinical examination. Testicular units were classified as atrophic when they were significantly smaller (typically ≥ 50% reduction in size compared to the contralateral testis) and exhibited either a soft consistency or a characteristically firm consistency, in combination with a small size (generally < 1–2 mL, depending on age). Complications were ranked according to the Clavien‐Dindo classification [17].

The institutional ethics committee approved the study and data extraction from patient records (ABR‐47524).

## 3 Results

A total of 254 orchidopexies were performed in 211 patients. Five patients, constituting 2% (5/254) of testicular units, were lost to follow‐up due to relocation abroad. Follow‐up rate was thus 98% (Table 2).

**TABLE 2:** Patient characteristics.

Procedure	Patients (*n*)	Testes (*n*)	Age	Follow‐up (*n*)
Inguinal orchidopexy	175	211	5.2 ± 4.2	206 (97.6%)
Laparoscopic (staged) orchidopexy	18	25	2.3 ± 3.6	25 (100%)
Redo‐orchidopexy	18	18	7.0 ± 4.1	18 (100%)
Total	211	254	5.1 ± 4.2	249 (98%)

*Note:* Age at time of surgery is expressed in years and presented as mean ± standard deviation. Number of testicular units reviewed postoperatively at follow up is presented per subgroup as number and percentage of total.

Abbreviation: *n*, number.

Short‐term complications were wound infections (4/249) and, in one case, injury of the vas deferens. The short‐term complication rate was calculated as 5/249 (2%). Table 3 gives an overview of short‐term complications stratified by type of surgery and complication grade according to the Clavien‐Dindo classification. One patient developed necrotizing fasciitis due to a streptococcal infection following an inguinal orchidopexy (classified as a grade IV complication). This rare complication was successfully treated with immediate surgery and admission to the intensive care. In the end, testicular volume was adequate, but the testicle was located in the inguinal canal.

**TABLE 3:** Short‐term complications.

Procedure	*n*	Number (%) of complications staged according to Clavien‐Dindo classification [17]				
		I	II	III	IV	V
Inguinal orchidopexy	206	1 (0.5%)	1 (0.5%)	1 (0.5%)	1 (0.5%)	0
Laparoscopic (staged) orchidopexy	25	0	0	0	0	0
Redo‐orchidopexy	18	0	0	1 (5.6%)	0	0
Total	249	1 (0.4%)	1 (0.4%)	2 (0.8%)	1 (0.4%)	0

Abbreviation: *n*, number of testicular units per operative procedure.

Median follow‐up was 7 months (interquartile range: 5–9 months) after the last operative procedure. Outcome was deemed satisfactory in 237/249 (95%) testicular units operated. Outcome at follow‐up per operative procedure is presented in Table 4. In four cases, the position of the testis was inguinal; 3 after inguinal orchidopexy and 1 following a laparoscopic staged Fowler Stephens procedure (which was ascribed to insufficient length of the testicular vessels). In total 8 (3.2%) testicular units were atrophied.

**TABLE 4:** Testicle position and volume at follow up stratified per procedure.

Procedure	*n*	Inguinal	Atrophy	Scrotal
Inguinal orchidopexy	206	3 (1.2%)	3 (1.2%)	200 (97.1%)
Laparoscopic (staged) orchidopexy	25	1 (4%)	4 (16%)	20 (80%)
Redo‐orchidopexy	18	0	1 (5.6%)	17 (94.4%)
Total	249	4 (1.6%)	8 (3.2%)	237 (95.2%)

*Note:* Position of the testis after surgery was deemed inguinal when the testis was in the inguinal canal and not (high in) scrotum.

Abbreviation: *n*, number of testicular units per operative procedure.

## 4 Discussion

In conclusion, in this single‐center, retrospective analysis of 249 orchidopexies, all performed under the supervision of or by fellowship‐trained pediatric urologists, the overall success rate was 95%. Short‐term complications consisted mostly of post‐operative infections and occurred in 2% of procedures. Loss to follow‐up in our study was minimalized by aggressively managing no‐shows at the outpatient, to no more than 2%. The reported outcomes can, therefore, be used in more accurate patient/parental counseling in daily clinical practice and clinical guidelines.

As of now, three randomized control trials of (the different surgical approaches to) orchidopexy have been published [18, 19, 20]. The vast majority of studies reporting on orchidopexy outcomes have predominantly been retrospective analyses of single‐center cohorts. In 2022, the American Pediatric Surgical Association published a systematic literature review concerning orchidopexy surgery outcome [8]. In the final analysis, the authors included 260 articles on a total of 5035 orchidopexies combined. The median participant number of these studies was 120 [18, 19, 20]. In short, the long‐term outcome of orchidopexies depends on the preoperative position of the NST (inguinal vs. abdominal), the procedure performed (open vs. laparoscopic) and whether the NST has been operated on previously (first attempt, staged vs. redo‐orchidopexy).

In our study, the success rate of all procedures combined was 95%. Procedures were deemed unsuccessful either due to atrophy or inadequate position (i.e., inguinal and not scrotal) of the operated testicle upon follow‐up. In two‐thirds of failed procedures (or 3.2% of all procedures) the outcome was deemed unsatisfactory due to atrophy. In literature, testicular atrophy following orchidopexy has been reported to range from 0% to 9% [8, 18, 21]. Inadequate position of the operated testicle upon follow‐up was the outcome in one‐third of unsuccessful procedures (1.6% of all procedures). With regard to inguinal position following orchidopexy, literature states an average rate of 1.3% for inguinal orchidopexy and a range of 1.5%–5% for orchidopexy in abdominally located testes [8]. Outcomes in our study, 1.2% and 4% respectively, were within these expectations. Finally, short‐term complications in our cohort were mostly of infectious nature. Infectious complications following orchidopexy surgery reported in literature have varied between 1% and 3% [22, 23]. The 2% complication rate in our study compares well with these findings.

In our cohort, outcomes were within known ranges when compared with success and complication rates previously reported in literature [8, 19, 20]. Most notably, our redo operation rates (8.5%) are comparable to recent cohorts (9.3%), which is an improvement compared to older cohorts (15%) [24]. This improved outcome over the years might be due to increased specialty training. In the future, outcomes might further be improved by honing existing techniques or even transitioning into others, such as single incision scrotal orchiopexy or staged traction orchiopexy [14, 25, 26, 27, 28].

Like previous studies into orchidopexy outcomes, ours was a retrospective analysis of a single center cohort. However, it stands out by its very high follow‐up rate of no less than 98%. Loss to follow‐up occurred only due to patients relocating to another country. This minimal loss allowed us to ascertain the actual complication rate of orchidopexy surgery, minimize bias, and thus improve the reliability of outcomes reported. These data can, in turn, be used both to inform patients and their parents and to improve clinical practice guidelines. Indeed, a recent report identified the lack of complete follow‐up data as a barrier to implementation and a possible area for improvement specifically of interest to guidelines on NST [29].

Our study has several limitations. One is its retrospective character, which can introduce bias and should be taken into account when considering the validity of the data. Second, data were obtained from a single surgical center, which may limit the generalizability of the results. Also, albeit that our study cohort size was well above the median number of patients in previous studies, attaining higher patient numbers would have allowed us to assess complication rates and outcomes better, especially within surgical technique subgroups and specifically within the staged orchidopexy group, which holds only 25 testicular units in 18 patients. Finally, our study reported on both inguinal orchidopexy and abdominal orchidopexy procedures combined, whereas most studies have reported on one treatment strategy only. We did, however, present outcomes for both procedures separately. It is our opinion that our report better reflects the common clinical practice of surgical centers where both surgical interventions are performed.

## Author Contributions

**Mick Uijldert:** investigation, formal analysis, visualization, writing – original draft, writing – review and editing. **Luitzen A. Groen:** formal analysis, supervision, conceptualization, methodology, visualization. **Keetje L. De Mooij:** conceptualization, writing – review and editing. **Michiel J. S. Oosterveld:** writing – original draft, writing – review and editing. **Harrie P. Beerlage:** writing – review and editing, supervision. **Tom P. V. M. De Jong:** conceptualization, visualization, supervision, writing – review and editing, investigation. **Rogier P. J. Schroeder:** writing – review and editing, supervision, conceptualization.

## Ethics Statement

Approval of the research protocol by an Institutional Reviewer Board: obtained (ABR‐47524).

## Consent

The authors have nothing to report.

## Conflicts of Interest

The authors declare no conflicts of interest.

## References

[1] K. A. Boisen , M. Kaleva , K. M. Main , et al., “Difference in Prevalence of Congenital Cryptorchidism in Infants Between Two Nordic Countries,” Lancet 363, no. 9417 (2004): 1264–1269, 10.1016/S0140-6736(04)15998-9. 15094270

[2] E. N. Bearrick , C. Dixon , A. Kaplan , et al., “Referral Patterns for Undescended Testis: A 7 Year Comparative Analysis of Primary Care Providers,” Journal of Pediatric Urology 17, no. 5 (2021): 736.e1–e6, 10.1016/j.jpurol.2021.06.017. 34736726

[3] D. L. Wenzler , D. A. Bloom , and J. M. Park , “What Is the Rate of Spontaneous Testicular Descent in Infants With Cryptorchidism?,” Journal of Urology 171, no. 2 Pt 1 (2004): 849–851, 10.1097/01.ju.0000106100.21225.d7. 14713841

[4] F. J. Schneuer , A. J. A. Holland , G. Pereira , S. Jamieson , C. Bower , and N. Nassar , “Age at Surgery and Outcomes of an Undescended Testis,” Pediatrics 137, no. 2 (2016): e20152768, 10.1542/peds.2015-2768. 26801912

[5] K. Williams , L. Baumann , A. Shah , F. Abdullah , E. K. Johnson , and T. A. Oyetunji , “Age at Orchiopexy for Undescended Testis in the United States,” Journal of Pediatric Surgery 53, no. 1 (2018): 86–89, 10.1016/j.jpedsurg.2017.10.020. 29102151

[6] K. Sijstermans , W. W. M. Hack , R. W. Meijer , and L. M. van der Voort‐Doedens , “The Frequency of Undescended Testis From Birth to Adulthood: A Review,” International Journal of Andrology 31, no. 1 (2008): 1–11, 10.1111/j.1365-2605.2007.00770.x. 17488243

[7] F. Hadziselimovic and B. Herzog , “The Importance of Both an Early Orchidopexy and Germ Cell Maturation for Fertility,” Lancet (London, England) 358 (2001): 9288, 10.1016/S0140-6736(01)06274-2. 11597673

[8] R. L. Gates , J. Shelton , K. A. Diefenbach , et al., “Management of the Undescended Testis in Children: An American Pediatric Surgical Association Outcomes and Evidence Based Practice Committee Systematic Review,” Journal of Pediatric Surgery 57, no. 7 (2022): 1293–1308, 10.1016/j.jpedsurg.2022.01.003. 35151498

[9] D. S. Engeler , P. O. Hösli , H. John , et al., “Early Orchiopexy: Prepubertal Intratubular Germ Cell Neoplasia and Fertility Outcome,” Urology 56, no. 1 (2000): 144–148, 10.1016/s0090-4295(00)00560-4. 10869645

[10] C. Radmayr , H. S. Dogan , P. Hoebeke , et al., “Management of Undescended Testes: European Association of Urology/European Society for Paediatric Urology Guidelines,” Journal of Pediatric Urology 12, no. 6 (2016): 335–343, 10.1016/j.jpurol.2016.07.014. 27687532

[11] C. Radmayr and S. Tekgul , “Paediatric Urology and the Dilemma of Low‐Quality Evidence for the Management of Common Urological Conditions (Vesicoureteral Reflux, Lower Urinary Tract Dysfunction, Undescended Testis) in Children,” European Urology Focus 3, no. 2–3 (2017): 308–309, 10.1016/j.euf.2017.03.012. 28753880

[12] L. H. Braga , A. J. Lorenzo , and R. L. P. Romao , “Canadian Urological Association‐Pediatric Urologists of Canada (CUA‐PUC) Guideline for the Diagnosis, Management, and Followup of Cryptorchidism,” Canadian Urological Association Journal 11, no. 7 (2017): E251–E260, 10.5489/cuaj.4585. 28761584 PMC5519382

[13] T. F. Kolon , C. D. A. Herndon , L. A. Baker , et al., “Evaluation and Treatment of Cryptorchidism: AUA Guideline,” Journal of Urology 192, no. 2 (2014): 337–345, 10.1016/j.juro.2014.05.005. 24857650

[14] M. Dayanc , Y. Kibar , H. C. Irkilata , E. Demir , L. Tahmaz , and A. F. Peker , “Long‐Term Outcome of Scrotal Incision Orchiopexy for Undescended Testis,” Urology 70, no. 4 (2007): 786–788, 10.1016/j.urology.2007.04.053. 17991558

[15] S. Bergbrant , E. Omling , J. Björk , and L. Hagander , “Cryptorchidism in Sweden: A Nationwide Study of Prevalence, Operative Management, and Complications,” Journal of Pediatrics 194 (2018): 197–203.e6, 10.1016/j.jpeds.2017.09.062. 29331326

[16] R. W. Meijer , W. W. M. Hack , L. M. Van Der Voort‐Doedens , K. Haasnoot , and S. D. Bos , “Surgical Findings in Acquired Undescended Testis,” Journal of Pediatric Surgery 39, no. 8 (2004): 1242–1244, 10.1016/j.jpedsurg.2004.04.016. 15300536

[17] D. Dindo , N. Demartines , and P. A. Clavien , “Classification of Surgical Complications: A New Proposal With Evaluation in a Cohort of 6336 Patients and Results of a Survey,” Annals of Surgery 240, no. 2 (2004): 205–213, 10.1097/01.sla.0000133083.54934.ae. 15273542 PMC1360123

[18] M. Nazem , M. Hosseinpour , and A. Alghazali , “Trans‐Scrotal Incision Approach Versus Traditional Trans‐Scrotal Incision Orchiopexy in Children With Cryptorchidism: A Randomized Trial Study,” Advanced Biomedical Research 8, no. 1 (2019): 34, 10.4103/abr.abr_26_19. 31259163 PMC6543866

[19] A. A. Elderwy , A. Kurkar , M. S. Abdel‐Kader , et al., “Laparoscopic Versus Open Orchiopexy in the Management of Peeping Testis: A Multi‐Institutional Prospective Randomized Study,” Journal of Pediatric Urology 10, no. 4 (2014): 605–609, 10.1016/j.jpurol.2014.06.006. 25042877

[20] W. M. Ghnnam , B. Saed , and H. Ghazy , “A Modified Technique for Scrotal Fixation During Orchiopexy,” African Journal of Paediatric Surgery : AJPS 8, no. 2 (2011): 203–205, 10.4103/0189-6725.86063. 22005366

[21] S. H. Ein , A. Nasr , P. W. Wales , and A. Ein , “Testicular Atrophy After Attempted Pediatric Orchidopexy for True Undescended Testis,” Journal of Pediatric Surgery 49, no. 2 (2014): 317–322, 10.1016/j.jpedsurg.2013.11.048. 24528976

[22] Y. S. Bassel , H. C. Scherz , and A. J. Kirsch , “Scrotal Incision Orchiopexy for Undescended Testes With or Without a Patent Processus Vaginalis,” Journal of Urology 177, no. 4 (2007): 1516–1518, 10.1016/j.juro.2006.11.075. 17382769

[23] P. R. H. Callewaert , M. S. Rahnama'i , B. T. Biallosterski , and P. E. V. van Kerrebroeck , “Scrotal Approach to Both Palpable and Impalpable Undescended Testes: Should It Become Our First Choice?,” Urology 76, no. 1 (2010): 73–76, 10.1016/j.urology.2009.09.096. 20156655

[24] J. Vikraman , S. Vidmar , S. Donath , and J. M. Hutson , “Frequency of Revision Orchidopexy in Australia 1995–2014,” Journal of Pediatric Surgery 52, no. 12 (2017): 1940–1943, 10.1016/j.jpedsurg.2017.08.061. 28964408

[25] C. Yu , Y. Hu , L. Wang , et al., “Comparison of Single‐Incision Scrotal Orchiopexy and Traditional Two‐Incision Inguinal Orchiopexy for Primary Palpable Undescended Testis in Children: A Systematic Review and Meta‐Analysis,” in Frontiers in Pediatrics, vol. 10 (Frontiers Media S.A., 2022), 805579, 10.3389/fped.2022.805579. 35372152 PMC8964791

[26] H. F. F. Novaes , J. A. C. Neto , A. Macedo , and U. Barroso , “Single Scrotal Incision Orchiopexy ‐ a Systematic Review,” International Brazilian Journal of Urology 39, no. 3 (2013): 305–311, 10.1590/S1677-5538.IBJU.2013.03.02. 23849581

[27] J. C. Hutcheson , C. S. Cooper , and H. M. Snyder , “The Anatomical Approach to Inguinal Orchiopexy,” Journal of Urology 164, no. 5 (2000): 1702–1704, 10.1016/S0022-5347(05)67088-7. 11025753

[28] S. Shehata , R. Shalaby , M. Ismail , M. Abouheba , and A. Elrouby , “Staged Laparoscopic Traction‐Orchiopexy for Intraabdominal Testis (Shehata Technique): Stretching the Limits for Preservation of Testicular Vasculature,” Journal of Pediatric Surgery 51, no. 2 (2016): 211–215, 10.1016/j.jpedsurg.2015.10.063. 26655212

[29] J. K. Kim , M. E. Chua , J. M. Ming , et al., “A Critical Review of Recent Clinical Practice Guidelines on Management of Cryptorchidism,” Journal of Pediatric Surgery 53, no. 10 (2018): 2041–2047, 10.1016/j.jpedsurg.2017.11.050. 29269095

